# Oxidized Oils and Oxidized Proteins Induce Apoptosis in Granulosa Cells by Increasing Oxidative Stress in Ovaries of Laying Hens

**DOI:** 10.1155/2020/2685310

**Published:** 2020-08-01

**Authors:** Ling Zhou, Xuemei Ding, Jianping Wang, Shiping Bai, Qiufeng Zeng, Zuowei Su, Yue Xuan, Aimin Wu, Keying Zhang

**Affiliations:** Institute of Animal Nutrition, Key Laboratory for Animal Disease-Resistance Nutrition of China Ministry of Education, Sichuan Agricultural University, 211 Huimin Road, Wenjiang, Chengdu, Sichuan, China 611130

## Abstract

The storage and preparation of corn for animal feed inevitably lead to lipid and protein peroxidation. Granulosa cells play an important role in follicular development in the ovaries, and hen laying productivity is likely to be dependent on follicle health and number. We hypothesized that oxidized oil and protein induce apoptosis via oxidative stress in laying hen granulosa cells. A sample of 360 38-week-old Lohmann commercial laying hens was used in a 2 × 2 factorial design for 8 weeks. Dietary treatments included dietary oil (fresh corn oil (FO) or oxidized corn oil (OO)) and corn gluten meal (fresh corn gluten meal (FP) or oxidized corn gluten meal (OP)). Productivity, ovarian histology, granulosa cell apoptosis, and indicators of oxidative stress were evaluated in all groups. Both dietary OO and OP decreased egg production and the average daily feed intake (ADFI) of laying hens. Flow cytometry, TUNEL, and real-time PCR revealed that both dietary OO and OP induced granulosa cell apoptosis in prehierarchical and hierarchical follicles. Furthermore, dietary OO and OP caused oxidative stress in prehierarchical and hierarchical follicles, as indicated by the downregulation of antioxidant-related-gene expression. Moreover, forkhead box O1 (FoxO1), extracellular regulated protein kinase (ERK), and c-Jun NH_2_ kinase (JNK) are involved in potential apoptosis regulation pathways in the granulosa cells of laying hens fed OO and OP, as indicated by the upregulation of FoxO1 expression and downregulation of ERK/JNK expression. These results indicate that OO and OP induce granulosa cell apoptosis via oxidative stress, and the combined use of OO and OP aggravates the adverse effects of oxidative stress in laying hens.

## 1. Introduction

Oils are often used in animal diets to increase their energy levels. Protein is known to be the most important component and is the main source of nitrogen, which monogastric animals use to synthesize proteins. However, both oils and proteins are often exposed to oxidants or oxidizing conditions during the preparation or storage of feeds [1, 2].

Feeding experiments involving animals revealed that the ingestion of oxidized fats and oxidized proteins provokes a wide array of biological effects [3–6], one of the most striking of which is the induction of oxidative stress due to the absorption of lipid hydroperoxide or advanced oxidation protein products [6–8]. Oxidized oil in animal feed was demonstrated to decrease the concentrations of exogenous and endogenous antioxidants and increase the activity of endogenous free radical scavenging enzymes and concentrations of lipid and protein peroxidation products [1, 7, 9, 10], thus decreasing the productivity of laying hens and broilers. A previous study reported that heat and malondialdehyde- (MDA-) oxidized wheat peptides show a relatively lower free radical scavenging activity, and the treatments also led to higher reactive oxygen species (ROS) production in rats [11]. The interactions between oxidized oils and oxidized proteins and whether oxidized proteins can cause similar damage to the productivity of laying hens are unclear.

Egg production may be regulated by the number of follicles. For example, the egg production rate was found to decrease with the age of hens, and older laying hens had more atretic follicles, resulting in fewer viable follicles and less egg production [12, 13]. Whether the follicle ultimately ovulates or undergoes atresia is dependent on the expression and actions of factors promoting follicular cell proliferation, growth, differentiation, and apoptosis [14]. Granulosa cells play an important role in follicular development. The apoptosis of granulosa cells leads to follicular atresia due to the insufficiency of survival signals and/or physiological or nonphysiological apoptotic signals [15]. Oxidative stress triggers granulosa cell apoptosis, which has been suggested to be a major cause of follicular atresia [16]. In addition, oxidative stress can trigger granulosa cell apoptosis through the forkhead box O 1 (FoxO1), phosphatidylinositol 3-kinase (PI3K), Akt, extracellular regulated protein kinase (ERK), and c-Jun NH_2_ kinase (JNK) pathways, thereby activating the transcription of either proapoptotic or stress-resistance genes [15, 17, 18].

A previous study reported a significant decrease in the productivity of laying hens fed oxidized oil [19]. Protein oxidation and lipid peroxidation usually interact with each other in feed [20]. Thereby, we hypothesized that oxidative stress, due to the presence of oxidized oils and proteins in feed, is involved in granulosa cell apoptosis We investigated the effects of oxidized oil, oxidized protein, or a combination on granulosa cell apoptosis elucidate possible mechanisms underlying the effects.

## 2. Material and Methods

### 2.1. Animal Care and Study Description

The study was approved by the Animal Care and Use Committee of Sichuan Agricultural University. The experiment was arranged in a 2 × 2 factorial design, and the main factors were 2% corn oil (fresh or oxidized) and 10% corn gluten meal (fresh or oxidized) in four diets: the control diet with fresh corn oil (FO) and fresh corn gluten meal (FP), the oxidized oil diet with oxidized corn oil (OO) and FP, the oxidized corn gluten meal (OP) diet with FO and oxidized corn gluten meal (OP), and the combination diet with both OO and OP. A total of 360 38-week-old Lohmann commercial laying hens were randomly allocated one of the four dietary treatments. There were six replicates for each treatment, and each replicate involved five cages with three hens/cage. The cages were 38.1 × 50 × 40 cm. Diets were formulated to meet Agricultural Trade Standardization of China [21] requirements for all nutrients (Table S1). Feed and water were supplied ad libitum, and a 16 h : 8 h light : dark photoperiod was used. The feeding trial lasted for 8 weeks.

### 2.2. Preparation of the Oxidized Oil and Oxidized Protein

The oxidized oil was prepared by heating corn oil (Huayu Co., Ltd., Chengdu, China) in an induction cooker (purchased online) for 56 d (6 h per day) at 100°C. Air was continuously bubbled throughout the oil heating process [22]. The extent of lipid peroxidation was determined by assaying the peroxide value [23] and acid concentrations [24]. The fatty acid composition of the dietary fats was determined by high-performance liquid chromatography.

The oxidized protein was prepared by dry heating corn gluten meal (EPPEN Biotechnology Co., Ltd., Ningxia, China) in an oven at 100°C for 24 h, which was then air-dried and stored at −20°C [1]. Samples were immediately collected for analysis of the amount of crude protein, protein carbonyl, and sulfhydryl in the fresh and heat-treated gluten meal. Protein carbonyl and sulfhydryl concentrations were measured using specific detection kits from Nanjing Jiancheng Bioengineering Institute (Nanjing, Jiangsu Province, P. R. China) with a Multiskan Spectrum Reader (Model 1500; Thermo Fisher Scientific, Nyon, Switzerland).

### 2.3. Sample Collection

Feed intake, egg production, and egg mass were determined daily, and the feed conversion rate (FCR) was calculated. Before slaughter, individual laying cycles were monitored for each hen throughout the laying sequence for 1 week. At week 8, 16 hens from each treatment were euthanized by cervical dislocation 7-9 h after the first or second oviposition of their egg sequence. Ovaries were removed under aseptic conditions, and images of the ovaries from each treatment were captured with a camera (EOS 80d, Canon group, Tokyo, Japan). Then, six ovaries from each treatment group were fixed in 4% paraformaldehyde for standard histological analysis (small yellow follicles and hierarchical follicle were excluded) and a terminal deoxynucleotide transferase dUTP nick-end labeling (TUNEL) assay of the prehierarchical and hierarchical follicles (*n* = 6). Six ovaries (small yellow follicles and hierarchical follicles were excluded) were stored at −20°C for use in an antioxidant enzyme activity assay (*n* = 6). The other ovaries were transferred to a Petri dish containing a physiological solution of PBS at 34°C, where they were sorted into distinct size categories (<1, 1-2, 2-3, 3-4, 4-5, and >5 mm) and counted. Granulosa cells from prehierarchical and hierarchical follicles (F5, F4, F3, F2, and F1) were separated, as previously described [25]. The harvested granulosa cells were divided, and one portion was used for quantitative analysis of apoptotic granulosa cells by flow cytometry and the other portion stored at −80°C for real-time PCR (*n* = 4).

### 2.4. Histological Assay

The 4% paraformaldehyde-fixed ovarian tissue (small yellow follicles and hierarchical follicle were excluded; *n* = 6) was embedded in paraffin, and 4 *μ*m thick sections were cut and mounted on microscope slides. The sections were deparaffinized in xylene and rehydrated using graded ethanol solutions, then stained with hematoxylin and eosin staining as described by Picut et al. [26].

### 2.5. RNA Extraction Real-Time PCR

Total RNA was isolated from prehierarchical and hierarchical follicles using Trizol reagent (Takara, Dalian, China), according to the manufacturer's protocol. The ratio of absorbance at 260 nm to that at 280 nm was calculated for each sample. The complementary DNA was synthesized using the primeScript RT reagent kit (Takara), and real-time PCR was performed using the SYBR Premix Ex Tap (Takara). The PCRs were run on an Applied Biosystems 7900HT Real-Time PCR system (Applied Biosystems, Foster City, CA, USA). All of these operations were conducted according to the manufacturers' instructions and Zhang et al. [27]. Amplification cycles involved initial heating at 95°C for 35 s, followed by 40 cycles of 95°C for 5 s, 60°C for 34 s, and 95°C for 15 s and an elongation step at 60°C for 15 s. Each mRNA level was expressed as its ratio to *β*-actin mRNA, and gene expression was calculated using the 2-*ΔΔ*Ct method [28]. All real-time PCRs were performed in triplicate in a 384-well plate. The sequences of the primers for apoptosis-related, antioxidant-related, and other genes are presented in Table S2.

### 2.6. Apoptosis Assay

#### 2.6.1. Flow Cytometry

Trypsin (Gibco, NY, USA) without EDTA was used to digest and collect all the samples. Flow cytometry was performed according to the apoptosis detection kit (Biolegend, 640932) procedures. Then, these cells incubated with 100 *μ*l binding buffer, containing 5 *μ*l APC-Annexin V apoptosis regent for 15 min on the ice. Next, 400 *μ*l binding buffer and 5 *μ*l of the propidium iodide (PI) mix were added before detecting. Flow cytometry was performed using a FACSCalibur flow cytometer (BD Biosciences, CA, USA).

#### 2.6.2. TUNEL Assay

The prehierarchical follicles (about 5 mm) and hierarchical follicles (about 1.5 cm) were randomly selected, embedded in paraffin, and serially sectioned into 4 *μ*m thick slices (*n* = 6). The apoptosis of serial sections was analyzed by TUNEL using the In Situ Cell Death Detection Kit according to the manufacturer's instructions (Roche, Shanghai, China). In brief, the tissue sections were washed in xylene and ethanol (absolute, 85%, and 75%, diluted in double-distilled water), suspended in PBS, and incubated for 25 min at 37°C with proteinase K (Servicebio Biotech, Wuhan, China). The slides were rinsed thrice in PBS, and the area around the sample was dried. Then, the TUNEL reaction mixture was added to the samples, which were incubated for 120 min at 37°C in a humidified atmosphere in the dark. DAPI (Servicebio Biotech, Wuhan, China) was then added for 10 min to label the nuclei. The apoptotic cells were analyzed using a fluorescent microscope (Leica DMI4000B, Wetzlar, Germany). At least ten images were collected for each replicate, and the number of TUNEL-positive granulosa cells was counted, and the average number of TUNEL-positive granulosa cells was calculated.

#### 2.6.3. Immunofluorescent and TUNEL Double Staining

Immunofluorescent and TUNEL double staining were used to localize the granulosa cells and identified as apoptotic. The prehierarchical and hierarchical follicles were treated and fixed as described for the TUNEL assay. The serial sections on slides were labeled with antibodies (rabbit polyclonal antibody, ABclonal Biotech, Wuhan, China) against follicle-stimulating hormone receptor (FSHR) overnight at 4°C, then washed with three changes of PBS prior to incubation with the appropriate fluorescently conjugated secondary antibody. Finally, the sections were washed in three changes of PBS, incubated with DAPI for 10 min in the dark, washed in deionized water, and mounted on glass slides with the hydrophilic Mowiol medium in glycerol-PBS (1 : 3, *v*/*v*). Cells were analyzed under a fluorescent microscope.

### 2.7. Antioxidant Status Assay

#### 2.7.1. Antioxidant Enzyme Activity

The ovarian tissue samples (*n* = 6) were homogenized in PBS and centrifuged at 4000 rpm for 10 min to obtain the supernatant. The activities of superoxide dismutase (SOD), glutathione peroxidase (GSH-Px), and catalase (CAT) and the MDA content were determined using specific detection kits (Nanjing Jiancheng Bioengineering Institute).

### 2.8. Statistical Analyses

Data were analyzed using ANOVA as a 2 × 2 factorial with GLM procedures of SPSS 23.0 (SPSS Inc., Chicago, IL). The main effects (oxidized corn oil or oxidized protein) and the interactions between the two factors were analyzed. Duncan's test was applied when any of the interactions showed significance. Data are shown as the means and pooled SEM. The results were considered significantly different at *P* values of ≤0.05.

## 3. Results

### 3.1. Characterization of the Experimental Corn Oils and Proteins

The experimental oils and proteins are outlined in Table 1. OO had the highest acid value (4.04 vs. 0.33 g KOH/kg) and peroxides value (7.6 vs. 4.0 mEq O_2_/kg). OP had the highest protein carbonyl (40.09 vs. 7.95 nmol/mg of protein).

### 3.2. Hen Performance

Weekly egg production, ADFI, FCR, and egg weight are shown in Table 2. Dietary OO significantly decreased egg production, ADFI, FCR, and egg weight in the whole period (*P* < 0.05) compared with the FO group. Similarly, dietary OP had significantly decreased the egg production by week 8 (*P* < 0.05, Figure S2) and the ADFI from week 5 to week 8 and the whole period (*P* < 0.05). There were OO × OP interaction effects observed on the FCR for the whole period and on the egg weight in weeks 5 to 8 (*P* < 0.05), in which the control group had lower FCR and the OO+FP group had a lower mean egg weight compared with the other groups.

### 3.3. Quantification of Follicles

The number of follicles of different sizes is shown in Table 3. There was no OO × OP interaction observed for the number of follicles, although the combination group had the fewest total follicles. Dietary OO significantly decreased (*P* < 0.05) the number of >5 mm follicles and the total number of follicles compared with the FP group. Dietary OP significantly decreased the number of 3-4 mm follicles (*P* < 0.05), and a trend (*P* = 0.06) was observed in the total number of follicles compared with the FP group.

### 3.4. Histological Assay

**Figure 1:**
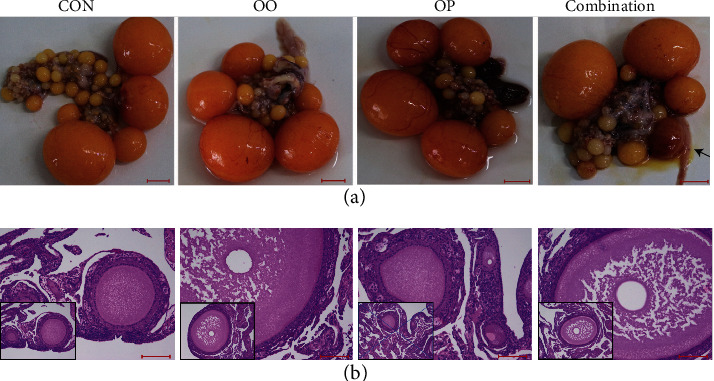
Effect of dietary oxidized oil and oxidized protein on ovary histology of laying hens. Images of the ovaries from each treatment were captured using a camera. Black arrows indicated atretic follicle (a, bar = 1 cm). Ovary histology was from ovarian tissue in which small yellow follicles and hierarchical follicle were excluded. Hematoxylin and eosin stain shows the appearance of ooplasm (bar = 100 *μ*m). The enclosed box in each panel shows the overall images (b). CON: control; OP: oxidized protein; OO: oxidized oil; Combination: oxidized oil+oxidized protein.

Images and photomicrographs of histology of the ovaries are shown in Figure 1. As shown in the images, here were abundant follicles in the control group, and atretic follicles were seen in the combination group. The ovaries of the control group and the OP group did not show structural differences. However, the ooplasm of the ovaries of the OO group was incomplete and gradually disappeared in contrast to that of the control and OP groups, which appeared normal.

### 3.5. Apoptosis Analysis

#### 3.5.1. Apoptosis Analysis by Flow Cytometry

**Figure 2:**
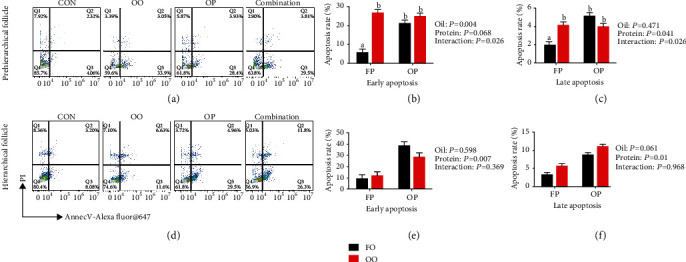
Effect of dietary-oxidized oil and dietary-oxidized protein on prehierarchical follicle (a–c) and hierarchical follicle (d–f) granulosa cell apoptosis rate of laying hens. Granulosa cells stained with fluorescein isothiocyanate- (APC-) Annexin V/propidium iodide (PI). Flow cytometric analysis defined early stage apoptosis as APC-Annexin V^+^ and PI^−^. Late stage apoptosis was defined as APC-Annexin V^+^ and PI^+^ in cells. Flow cytometry profiles with APC-Annexin V (*x*-axis) and PI (*y*-axis) staining (a, d). Calculated proportion of cells undergoing apoptosis (b, c, e, f). Values are the means ± SEMs (*n* = 4). Means without a common letter are different; *P* < 0.05. CON: control; FP: fresh protein; OP: oxidized protein; FO: fresh oil; OO: oxidized oil; Combination: oxidized oil+oxidized protein.

The quantification of the percentage apoptosis of granulosa cells by flow cytometry is illustrated in Figure 2 (*n* = 4). Dietary OO significantly increased the early apoptosis of granulosa cells of the prehierarchical follicles (*P* < 0.01) compared with the FO group. Dietary OP significantly increased the late apoptosis in granulosa cells from prehierarchical follicles (*P* < 0.05) and the early and late apoptosis of granulosa cells from hierarchical follicles (*P* < 0.05) compared with the FP group. There was an OO × OP interaction effect on the early and late apoptosis of granulosa cells from prehierarchical follicles, and the control group had showed the lowest levels of apoptosis of all the groups (*P* < 0.05).

#### 3.5.2. Apoptosis Analysis by TUNEL Assay

**Figure 3:**
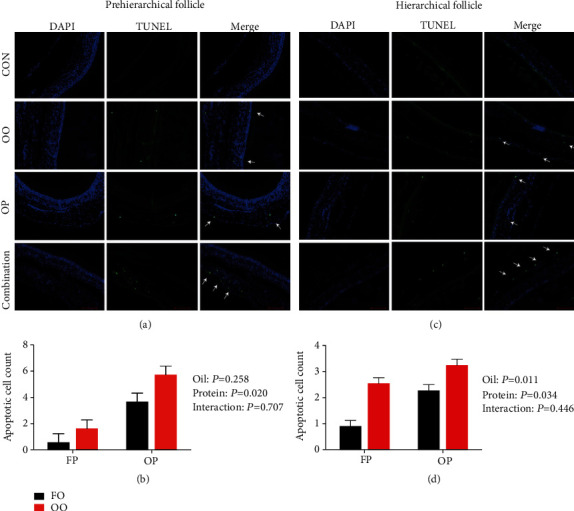
Effect of dietary oxidized oil and oxidized protein on levels of apoptotic granulosa cells in prehierarchical follicles (a, b) and hierarchical follicles (c, d) granulosa cells of laying hens. Nuclei were blue (DAPI). White arrows indicated TUNEL-positive granulosa cells (green) and quantified (b, d) in at least ten images (bar = 100 *μ*m). Values are the means ± SEMs (*n* = 4). CON: control; FP: fresh protein; OP: oxidized protein; FO: fresh oil; OO: oxidized oil; Combination: oxidized oil+oxidized protein.

The results of the TUNEL assays of the granulosa cells are shown in Figure 3. Analyses revealed there were very few TUNEL-positive cells in the control group. Both dietary OO- and OP induced apoptosis in granulosa cells from prehierarchical and hierarchical follicles, as indicated by the increased abundance of TUNEL-positive nuclei in these cells and the average number of TUNEL-positive granulosa cells in the experimental groups compared with the control group.

#### 3.5.3. Apoptosis Analysis by Immunofluorescence and TUNEL Double Staining

Figure S1 shows photomicrographs of immunofluorescence and TUNEL double-stained granulosa cells. The results of the immunofluorescence and TUNEL double staining were consistent with our TUNEL assay results. Both dietary OO and OP induced apoptosis in granulosa cells from prehierarchical and hierarchical follicles and granulosa cells double-stained with the TUNEL-positive cells.

#### 3.5.4. Apoptosis-Related Gene Expression

**Figure 4:**
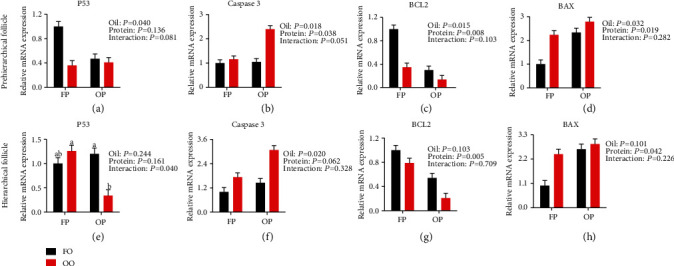
Effect of dietary oxidized oil and oxidized protein on the expression of apoptosis-related genes in prehierarchical follicles (a–d) and hierarchical follicles (e–h) granulosa cells of laying hens. Values are the means ± SEMs (*n* = 4). All the data were acquired using real-time PCR. Means without a common letter are different; *P* < 0.05. FP: fresh protein; OP: oxidized protein; FO: fresh oil; OO: oxidized oil.

The results of the analysis of apoptosis-related genes expression of granulosa cells are shown in Figure 4. Dietary OO downregulated (*P* < 0.05) the expression of P53 and BCL2 in prehierarchical follicle and upregulated (*P* < 0.05) the expression of caspase 3 and BAX in prehierarchical follicle and caspase 3 in hierarchical follicle compared with the FO group. Dietary OP reduced (*P* < 0.05) the expression of BCL2 in prehierarchical and hierarchical follicles and increased (*P* < 0.05) the expression of caspase 3 and BAX in prehierarchical follicles and BAX in hierarchical follicles compared with the FP group. There was an OO × OP interaction effect observed on hierarchical follicle P53 expression, in which hierarchical follicles in the combination group had the lowest expression levels of P53 of all the groups (*P* < 0.05).

#### 3.5.5. FoxO1 and JNK/ERK Genes Expression

**Figure 5:**
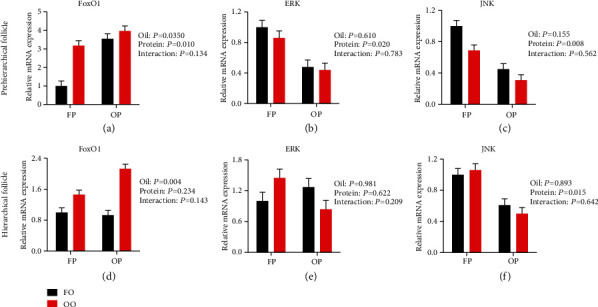
Effect of dietary oxidized oil and oxidized protein on the expression of FoxO1 (a, d), ERK (b, e), and JNK (c, f) in prehierarchical follicle and hierarchical follicle granulosa cells of laying hens. Values are the means ± SEMs (*n* = 4). All the data were acquired using real-time PCR. Means without a common letter are different; *P* < 0.05. FP: fresh protein; OP: oxidized protein; FO: fresh oil; OO: oxidized oil.

The results of the analysis of FoxO1 and JNK/ERK gene expression in granulosa cells are shown in Figure 5. Both dietary OO and OP upregulated (*P* < 0.05) prehierarchical follicle granulosa cell FoxO1 expression compared with the expression in the FO and FP groups, respectively. Dietary OP downregulated (*P* < 0.05) prehierarchical follicle JNK/ERK and hierarchical follicle JNK expression compared with the expression in the FP group.

### 3.6. Antioxidant Index Analysis

#### 3.6.1. Antioxidant Indexes

**Figure 6:**
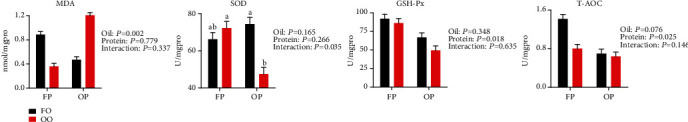
Effect of dietary oxidized oil and oxidized protein on ovary antioxidant enzyme activity of laying hens. Antioxidant enzyme activity assay was performed in the ovary in which small yellow follicles and hierarchical follicle were excluded. Values are the means ± SEMs (*n* = 6). Means without a common letter are different; *P* < 0.05. FP: fresh protein; OP: oxidized protein; FO: fresh oil; OO: oxidized oil; MDA: malondialdehyde; SOD: superoxide dismutase; GSH-Px: glutathione peroxidase; T-AOC: total antioxidant capacity.

The antioxidant activity of the ovaries is shown in Figure 6. Dietary OO significantly increased the MDA content (*P* < 0.05) of ovaries compared with the FO group, whereas dietary OP significantly (*P* < 0.05) decreased the GSH-Px activities and T-AOC compared with the FP group. There was an OO × OP interaction effect seen for SOD activity (*P* < 0.05) in which the combination group had significantly lower SOD activity.

#### 3.6.2. Antioxidant-Related Genes Expression

**Figure 7:**
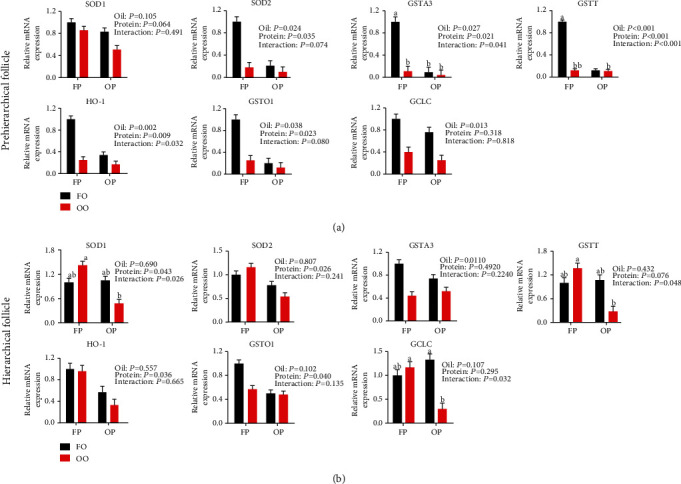
Effect of dietary oxidized oil and oxidized protein on the expression of antioxidant-related genes in prehierarchical follicle (a) and hierarchical follicle (b) granulosa cells of laying hens. Values are the means ± SEMs (*n* = 4). All the data were acquired using real-time PCR. Means without a common letter are different; *P* < 0.05. FP: fresh protein; OP: oxidized protein; FO: fresh oil; OO: oxidized oil.

The results of the analyses of antioxidant-related genes expression in granulosa cells are provided in Figure 7. Both OO and OP downregulated (*P* < 0.05) the expression of SOD2, glutathione S-transferase-A3 (GSTA3), glutathione transferase omega 1 (GSTO1), glutathione S-transferase theta (GSTT), and heme oxygenase-1 (HO-1) in prehierarchical follicles compared with expression in the FO and FP groups, respectively. Dietary OP downregulated (*P* < 0.05) the expression of SOD1, SOD2, GSTO1, and HO-1 in hierarchical follicles compared with that in the FP group. There were OO × OP interaction effects on the prehierarchical follicle expression of GSTA3, GSTT and HO-1, and the hierarchical follicle expression of SOD1, glutathione cysteine ligase catalytic (GCLC), and GSTT (*P* < 0.05).

## 4. Discussion

In the current study, both dietary OO and OP repressed the egg production of laying hens. This is consistent with the findings of some other studies in which dietary OO and OP were found to decrease the productivity of laying hens and broilers [11, 19, 29, 30]. Lipid and protein peroxidation products are produced during the heating of oils and proteins. Birds are easily exposed to cumulative oxidative byproducts when OO and OP are consumed, resulting in an impaired redox balance and lower productivity. These results also indicated that it takes longer for protein peroxidation products to accumulate and have adverse effects on the body than for lipid peroxidation products. The quality of the follicular directly determines the egg production rate of laying hens, and the egg production rate has been reported to strongly and negatively correlate with the incidence of follicle atresia [31]. In this study, we found evidence that both OO and OP induce follicular atresia. First, both OO and OP were found to decrease the follicular numbers, and atretic follicles were observed in the combination group. Second, the ooplasm in the ovaries of the OO group was incomplete, which may be a sign of oocyte degeneration [32]. In laying hens, more than 30% of the yolk mass is comprised of lipids imported as part of lipoproteins, mainly very low density lipoprotein and vitellogenin [33]. Dietary oxidized oil reduced the concentrations of cholesterol, triacylglycerol, and very-low density lipoprotein in the liver and plasma of animals [4], which decreases the importation of lipids into the follicles and leads to uneven appearance of ooplasm.

Hen follicular atresia is mediated by apoptosis, and a large majority of the cells undergoing this process appear to be of granulosa cell origin [34]. Flow cytometry, TUNEL, and immunofluorescent and TUNEL double staining analyses were performed to evaluate the effects of OO and OP on the apoptosis of laying hen ovarian granulosa cells. The results showed that both OO and OP can induce apoptosis in granulosa cells of prehierarchical follicle and hierarchical follicles, followed by an elevation in proapoptosis gene expression and a reduction in antiapoptosis gene expression.

Oxidative stress is considered the major drive behind granulosa cell apoptosis when laying hens are exposed to OO and OP. Accumulating evidence shows that oxidative stress causes the initiation of granulosa apoptosis, which leads to follicular atresia [18, 35, 36]. The exposure of rat granulosa cells to sodium fluoride and T-2 toxin induced granulosa cell apoptosis through oxidative stress [18, 37]. In addition, hexavalent chromium can induce the apoptosis of spontaneously immortalized rat granulosa cells by increasing intraovarian ROS levels and depleting antioxidant enzymes, and vitamin C supplementation alleviates the adverse effects by increasing the antioxidant capacity of the cells [38]. During the heating of corn oil and corn protein, lipids are oxidized to produce hydroperoxides, and proteins are oxidized to produce advanced oxidation protein products, resulting in various levels of oxidants in feed [39–41]. When ingestion of the excess oxidants overwhelms the cellular antioxidant defense system, oxidative stress occurs [42]. In our study, both dietary OO and OP inhibited SOD and GSH-Px activities and decrease T-AOC in ovaries of laying hens, which resulted in the excessive production of ROS and subsequent oxidative stress. We measured the expression of the antioxidant genes SOD1, SOD2, GCLC, GSTT, GSTA3, GSTO1, and HO-1 in granulosa cells. SOD1 and SOD2 are two intracellular enzymes responsible for the dismutation of superoxide to H_2_O_2_ in the ovaries [36]. HO-1 and GSTs work in synergy to provide pleiotropic cellular defense, scavenging ROS and detoxifying electrophiles [43]. In general, our results indicate that OO and OP caused an accumulation of superoxides and a decrease in the antioxidant capacity of the system, which further induced oxidative stress and inhibited follicular growth. Moreover, protein oxidation and lipid peroxidation usually interact with each other in feed [20]. The interactions of GSTA3, GSTT, HO-1, and GCLC indicated that the combined use of OO and OP aggravated the adverse effects of laying hens. In addition, the number of unhealthy follicles has been reported to increase with decreasing follicle size [13]. Our results support the view that the adverse effects of laying hens fed OO and OP are more severe in prehierarchical follicles than in hierarchical follicles.

Furthermore, our data demonstrated that both OO and OP upregulated the expression of FoxO1 and OP downregulated the expression of JNK/ERK. FoxOs, a subfamily of transcription factors, are able to regulate diverse cellular processes, such as DNA damage, stress response, and metabolism. In apoptotic granulosa cells, FoxOs are dephosphorylated and translocated to the nucleus, resulting in the enhancement of the proapoptotic factors transcription [44]. In mouse granulosa cells, oxidative stress increased the expression of FoxO1, and FoxO1 is known to target proapoptosis and antiapoptosis genes [17]. The mitogen activated protein kinase (MAPK) and PI3K signaling pathways have been shown to play crucial roles in oxidative stress-mediated apoptosis [45, 46]. It has been reported that H_2_O_2_ could induce apoptosis in bovine carotid endothelial cells and raloxifene analogue LY117018 could inhibit apoptosis by activating ERK1/2 [47]. Therefore, we measured the expression of FoxO1 and the MAPK family members, ERK and JNK, in granulosa cells. Our results suggested that FoxO1, ERK, and JNK are involved in apoptosis regulation pathways in the granulosa cells of laying hens fed OO and OP.

## 5. Conclusion

Our results suggest that dietary oxidized oil with an acid value of 4.04 g KOH/kg and oxidized protein with a protein carbonyl content of 40.09 nmol/mg of protein suppress the productivity of laying hens. These effects were ascribed to the induction of apoptosis in the prehierarchical and hierarchical follicles of laying hens. Oxidative stress plays a critical role in the apoptosis process, as indicated by a decrease in antioxidant enzyme activity and antioxidant-related gene expression. The combined use of oxidized oil and oxidized protein aggravates the adverse effects of oxidative stress on laying hens, as indicated by the interactions of antioxidant enzyme and antioxidant-related gene expression. FoxO1 and ERK/JNK are involved in potential apoptosis regulation pathways in the granulosa cells of laying hens fed oxidized oil and oxidized protein.

## Figures and Tables

**Table 1:** Characteristics of the experimental oil and protein used.

Corn oil			Corn gluten meal		
Item	Fresh oil	Oxidized oil	Item (g/kg)	Fresh protein	Oxidized protein
Acid value (g KOH/kg)	0.33	4.04	Dry matter	910.2	985.9
Peroxides (mEq O_2_/kg)	4.0	7.6	Crude protein	639.1	647.4
Fatty acid composition (g fatty acid/100 g total fatty acids)			Crude fat	50.3	31.0
16 : 0	12.57	15.39	Protein carbonyl (nmol/mg of protein)	7.95	40.09
18 : 0	1.67	2.07	Free sulfhydryl (nmol/mg of protein)	7.80	4.39
18 : 1	27.78	32.00	Total disulfide and sulfhydryl (nmol/mg of protein)	65.22	50.72
18 : 2 (n-6)	55.53	47.26			
18 : 3 (n-3)	0.79	0.48			

**Table 2:** Effect of dietary oxidized oil and oxidized protein on productivity in laying hens^1, 2^.

Item	Time (wk)	Fresh oil		Oxidized oil		Pooled SEM	*P* value		
		Fresh protein	Oxidized protein	Fresh protein	Oxidized protein		Oil	Protein	Interaction
Egg production (%)	1-4	86.87	85.95	85.45	82.64	0.885	0.197	0.305	0.597
	5-8	89.42	86.19	84.13	83.04	0.782	0.014	0.182	0.503
	1-8	88.14	86.09	84.62	82.83	0.779	0.042	0.232	0.934
ADFI (g/hen/day)	1-4	106.92	106.63	105.30	105.60	0.196	0.003	0.990	0.460
	5-8	110.01	107.88	108.62	106.15	0.403	0.067	0.010	0.835
	1-8	108.43	107.24	106.96	105.89	0.266	0.016	0.046	0.911
FCR	1-4	2.01	2.12	2.20	2.20	0.022	0.007	0.247	0.284
	5-8	2.01	2.07	2.16	2.11	0.018	0.015	0.809	0.150
	1-8	2.03^b^	2.10^a,b^	2.15^a^	2.14^a,b^	0.017	0.039	0.607	0.006
Egg weight (g)	1-4	59.67	58.65	57.53	58.99	0.203	0.076	0.911	0.106
	5-8	61.33^a^	60.68^a,b^	59.87^b^	60.61^a,b^	0.204	0.038	0.728	0.018
	1-8	60.50	59.65	58.66	59.78	0.191	0.03	0.461	0.297

^1^Each value represents the mean values of 6 replicates (*n* = 6). ^2^Means without a common letter are different; *P* < 0.05. SEM: standard error of the mean; ADFI: average daily feed intake; FCR: feed converse rate.

**Table 3:** Effect of dietary oxidized oil and oxidized protein on follicular numbers in laying hens^1^.

Follicular size (mm)	Fresh oil		Oxidized oil		Pooled SEM	*P* value		
	Fresh protein	Oxidized protein	Fresh protein	Oxidized protein		Oil	Protein	Interaction
<1	158.50	122.25	106.50	114.50	8.741	0.113	0.435	0.230
1-2	44.00	41.00	40.50	29.50	3.193	0.263	0.295	0.543
2-3	18.50	14.00	16.75	17.00	1.904	0.872	0.587	0.544
3-4	14.00	7.75	9.25	5.00	0.871	0.052	0.011	0.576
4-5	7.75	8.00	5.25	3.50	0.849	0.062	0.667	0.567
>5	21.00	17.50	14.50	14.75	0.943	0.030	0.406	0.340
Total	263.75	210.50	192.75	184.25	7.494	0.007	0.062	0.161

^1^Each value represents the mean values of 6 replicates (*n* = 6). ^2^SEM: standard error of the mean.

## Data Availability

The data used to support the findings of this study is available from the corresponding author upon request.

## Supplementary Materials

Supplementary 1 Figure S1: apoptotic granulosa cells in prehierarchical follicle and hierarchical follicle granulosa cells of laying hens.

Supplementary 2 Table S1: diet composition and nutrient levels.

Supplementary 3 Table S2: primers used for quantitative real-time PCR analysis.

Supplementary 4 Figure S2: egg production curve from 1 to 8 weeks.
