# Supplementary material for: Oxidized Oils and Oxidized Proteins Induce Apoptosis in Granulosa Cells by Increasing Oxidative Stress in Ovaries of Laying Hens
Source: Oxid Med Cell Longev. 2020 Aug 1;2020:2685310. doi: 10.1155/2020/2685310 (PMC7422066; doi:10.1155/2020/2685310)
Supplement: Supplementary 1 — Figure S1: apoptotic granulosa cells in prehierarchical follicle and hierarchical follicle granulosa cells of laying hens. [file 2685310.f1.docx]

**
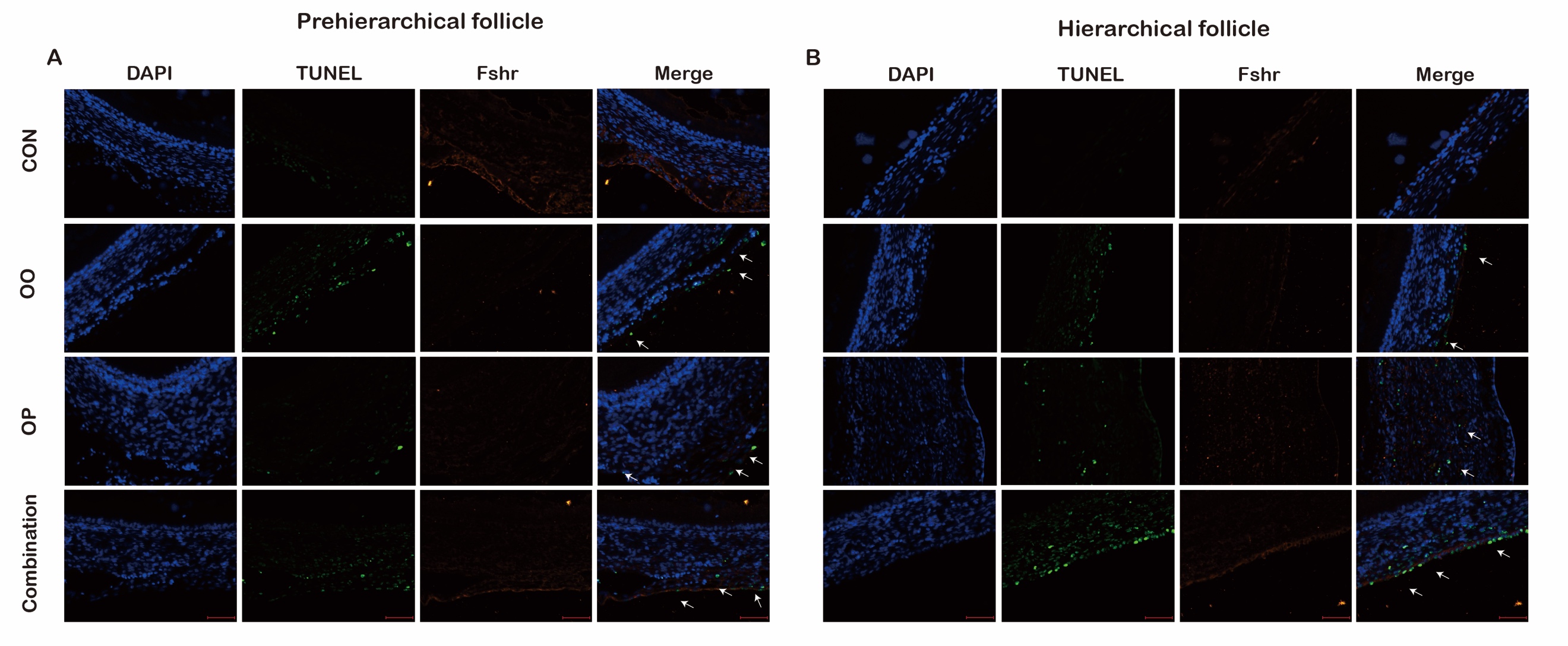
**

**Figure S1** Effect of dietary OO and OP on levels of apoptotic granulosa cells in prehierarchical follicles (*A*) and hierarchical follicles (*B*) granulosa cells of laying hens. Nuclei were blue (DAPI). FSHR-positive were red. White arrows indicated TUNEL-positive granulosa cells (green, Bar = 100 µm). Fshr, follicle stimulating hormone receptor; CON, control; FP, fresh protein; OP, oxidized protein; FO, fresh oil; OO, oxidized oil; Combination, oxidized oi + oxidized protein.
