# Supplementary material for: Oxidized Oils and Oxidized Proteins Induce Apoptosis in Granulosa Cells by Increasing Oxidative Stress in Ovaries of Laying Hens
Source: Oxid Med Cell Longev. 2020 Aug 1;2020:2685310. doi: 10.1155/2020/2685310 (PMC7422066; doi:10.1155/2020/2685310)
Supplement: Supplementary 2 — Table S1: diet composition and nutrient levels. [file 2685310.f2.docx]

**Supplementary Table 1**. **Diet composition and nutrient levels**

| Item | Content |
| --- | --- |
| Ingredient (g/kg) |  |
| Corn | 383.0 |
| Soybean meal | 132.0 |
| Corn starch | 150.0 |
| Corn gluten meal | 100.0 |
| Corn oil | 20.0 |
| Wheat bran | 80.0 |
| Rice bran with hull | 23.7 |
| Calcium carbonate | 84.0 |
| Calcium hydrophosphate | 12.6 |
| L-Lysine-HCl | 3.0 |
| DL-Methionine | 0.4 |
| Threonine  Tryptophan | 0.4 |
|  | 0.6 |
| Sodium chloride | 4.0 |
| Mineral premix^1^ | 5.0 |
| Vitamin premix^2^ | 0.3 |
| Choline chloride, 50% | 1.0 |
| Nutrient content^3^ |  |
| ME(Mj/kg) | 11.32 |
| Crude protein(%) | 16.55 |
| Calcium(%) | 3.53 |
| Available phosphorus (%) | 0.32 |
| Lysine(%) | 0.76 |
| Methionine(%) | 0.34 |
| Threonine(%) | 0.55 |
| Tryptophan(%) | 0.16 |

^1^mineral premix provided the following components per kilogram of the diet: iron, 60mg; copper, 8 mg; manganese, 60mg; zinc, 80mg; selenium, 0.3mg; iodine, 0.35mg.

^2^The vitamin premix provided the following components per kilogram of the diet: vitamin A, 8000IU; vitamin D_3_, 1600 IU, vitamin E, 5 IU; vitamin B_1,_ 0.8 mg; vitamin B_2_, 2.5 mg; vitamin B_6_, 1.5 mg; vitamin B_12_, 0.004 mg; D-pantothenic acid, 2.2 mg; folic acid, 0. 25 mg; nicotinic acid, 20 mg; biotin, 0.1 mg.

^3^Calculated value
