# Supplementary material for: Oxidized Oils and Oxidized Proteins Induce Apoptosis in Granulosa Cells by Increasing Oxidative Stress in Ovaries of Laying Hens
Source: Oxid Med Cell Longev. 2020 Aug 1;2020:2685310. doi: 10.1155/2020/2685310 (PMC7422066; doi:10.1155/2020/2685310)
Supplement: Supplementary 3 — Table S2: primers used for quantitative real-time PCR analysis. [file 2685310.f3.docx]

**Supplementary Table 2** Gene-specific primers for real-time quantitative reverse transcription PCR.

| Genes | Primers (5′ to 3′) | Genes number^α^ | Product size, bp |
| --- | --- | --- | --- |
| *p53* | Forward: GAGATGCTGAAGGAGATCAATGAG | NM_205264.1 | 145 |
|  | Reverse: GTGGTCAGTCCGAGCCTTTT |  |  |
| *BAX* | Forward: GTGATGGCATGGGACATAGCTC | XM_015290060.2 | 90 |
|  | Reverse: TGGCGTAGACCTTGCGGATAA |  |  |
| *Caspase-3* | Forward: TGGCCCTCTTGAACTGAAAG | [XM_015276122.2](https://www.ncbi.nlm.nih.gov/entrez/viewer.fcgi?db=nucleotide&id=1390106487) | 138 |
|  | Reverse: TCCACTGTCTGCTTCAATACC |  |  |
| *Bcl-2* | Forward: GATGACCGAGTACCTGAACC | [NM_205339.2](https://www.ncbi.nlm.nih.gov/entrez/viewer.fcgi?db=nucleotide&id=758371883) | 114 |
|  | Reverse: CAGGAGAAATCGAACAAAGGC |  |  |
| *SOD1* | Forward: TTGTCTGATGGAGATCATGGCTTC | [NM_205064.1](https://www.ncbi.nlm.nih.gov/entrez/viewer.fcgi?db=nucleotide&id=45384217) | 98 |
|  | Reverse: TGCTTGCCTTCAGGATTAAAGTGAG |  |  |
| *SOD2* | Forward: CAGATAGCAGCCTGTGCAAATCA | [NM_204211.1](https://www.ncbi.nlm.nih.gov/entrez/viewer.fcgi?db=nucleotide&id=45383701) | 86 |
|  | Reverse: GCATGTTCCCATACATCGATTCC |  |  |
| *GSTO1* | Forward: CATGATGTGGCCCTGGTTTG | [XM_015288649.2](https://www.ncbi.nlm.nih.gov/entrez/viewer.fcgi?db=nucleotide&id=1390116079) | 101 |
|  | Reverse: CAGTGCTGGAGCTTTGGAGTATGA |  |  |
| *HO-1* | Forward: TTGGCAAGAAGCATCCAGA | [NM_205344.1](https://www.ncbi.nlm.nih.gov/entrez/viewer.fcgi?db=nucleotide&id=45384397) | 214 |
|  | Reverse: TCCATCTCAAGGGCATTCA |  |  |
| *GCLC* | Forward: GGACAGGCACAGACACAGAA | [XM_419910.5](https://www.ncbi.nlm.nih.gov/entrez/viewer.fcgi?db=nucleotide&id=1390101177) | 232 |
|  | Reverse: TGCTGTGCGATGAATTCCCT |  |  |
| *GSTA3* | Forward: TTGGATAAGGCCGCAAACAGATA | [NM_001001777.1](https://www.ncbi.nlm.nih.gov/entrez/viewer.fcgi?db=nucleotide&id=49169815) | 115 |
|  | Reverse: TTTCCAGTAAATGCACGTCTGCTA |  |  |
| *GSTT* | Forward: GACGGAGACTTCACCCTAGCAGA | [NM_205365.1](https://www.ncbi.nlm.nih.gov/entrez/viewer.fcgi?db=nucleotide&id=45382478) | 87 |
|  | Reverse: TGATGGGTACCAGTGGTCAGGA |  |  |
| *FoxO1* | Forward: TCTGGTCAGGAGGGAAATGG | [NM_204328.1](https://www.ncbi.nlm.nih.gov/entrez/viewer.fcgi?db=nucleotide&id=45383501) | 60 |
|  | Reverse: GCTTGCAGGCCACTTTGAG |  |  |
| *ERK* | Forward: AGCAAGCTTTAGCCCATCCA | [XM_015275131.2](https://www.ncbi.nlm.nih.gov/entrez/viewer.fcgi?db=nucleotide&id=1390075115) | 108 |
|  | Reverse: CCTTCGGCAAGTCATCCAAT |  |  |
| *JNK* | Forward: GCATCCATCTTCGTCGTCAT | [XM_025151966.1](https://www.ncbi.nlm.nih.gov/entrez/viewer.fcgi?db=nucleotide&id=1390117891) | 121 |
|  | Reverse: TCATCTACAGCAACCCAGAGG |  |  |
