# Supplementary material for: Oxidized Oils and Oxidized Proteins Induce Apoptosis in Granulosa Cells by Increasing Oxidative Stress in Ovaries of Laying Hens
Source: Oxid Med Cell Longev. 2020 Aug 1;2020:2685310. doi: 10.1155/2020/2685310 (PMC7422066; doi:10.1155/2020/2685310)
Supplement: Supplementary 4 — Figure S2: egg production curve from 1 to 8 weeks. [file 2685310.f4.docx]

**
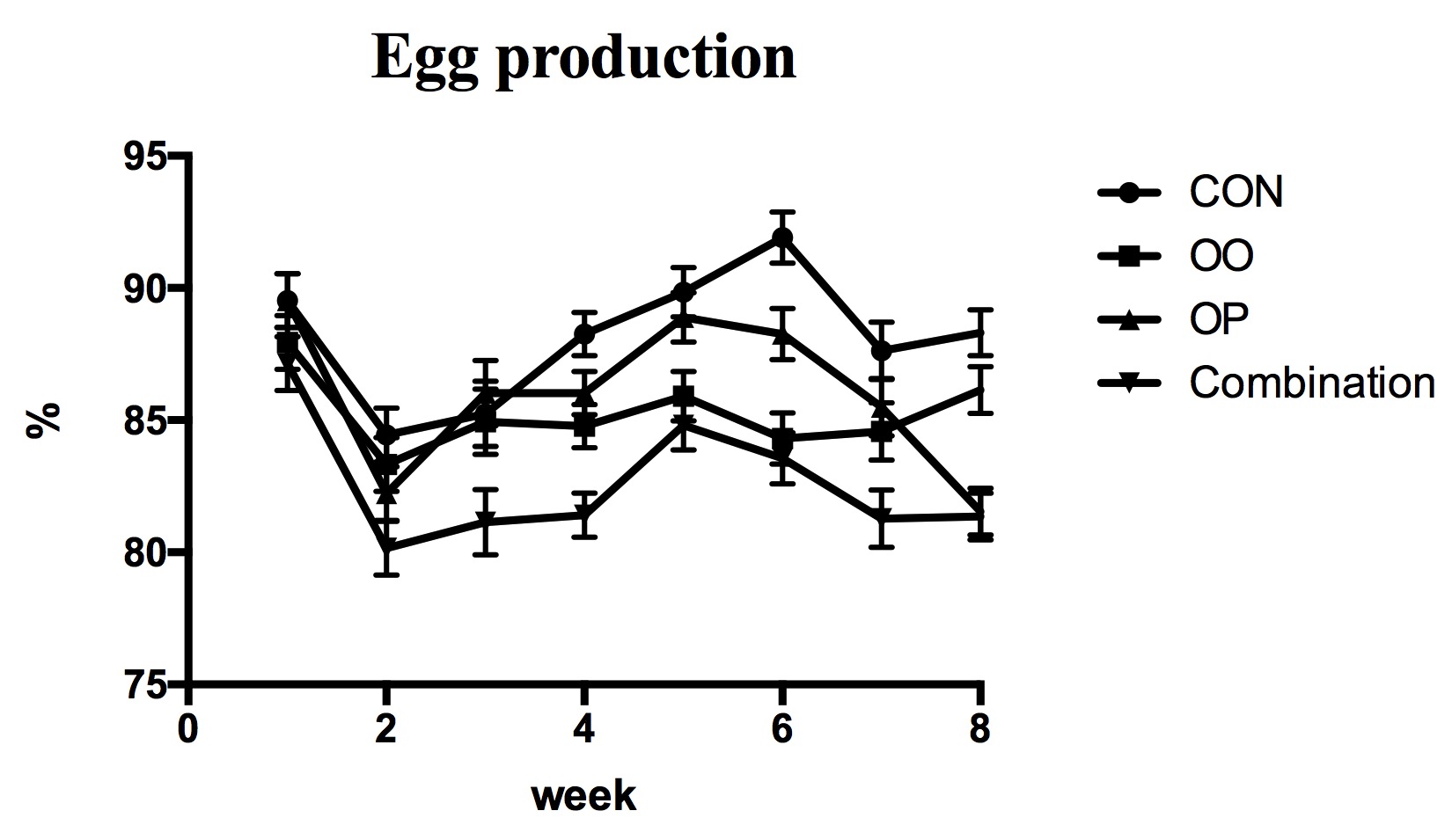
**

| Time  (wk) | *P* value | | |
| --- | --- | --- | --- |
|  | Oil | Protein | Interaction |
| 1 | 0.345 | 0.849 | 0.849 |
| 2 | 0.598 | 0.134 | 0.640 |
| 3 | 0.307 | 0.550 | 0.364 |
| 4 | 0.036 | 0.154 | 0.891 |
| 5 | 0.076 | 0.783 | 0.824 |
| 6 | 0.005 | 0.271 | 0.464 |
| 7 | 0.112 | 0.228 | 0.790 |
| 8 | 0.516 | 0.004 | 0.582 |

**Supplementary Figure 2. Egg Production Curve from 1 to 8 week.**
